# Automatic emotion recognition in healthcare data using supervised machine learning

**DOI:** 10.7717/peerj-cs.751

**Published:** 2021-12-15

**Authors:** Nazish Azam, Tauqir Ahmad, Nazeef Ul Haq

**Affiliations:** 1 Department of Computer Science, University of Engineering and Technology Lahore, Lahore, Pakistan; 2 School of Electrical Engineering and Computer Science, National University of Sciences and Technology, Islamabad, Pakistan

**Keywords:** Emotion detection, Emotion guidance scale, Negative emotions mapping, Supervised machine learning, Patient’s emotion

## Abstract

Human feelings are fundamental to perceive the conduct and state of mind of an individual. A healthy emotional state is one significant highlight to improve personal satisfaction. On the other hand, bad emotional health can prompt social or psychological well-being issues. Recognizing or detecting feelings in online health care data gives important and helpful information regarding the emotional state of patients. To recognize or detection of patient’s emotion against a specific disease using text from online sources is a challenging task. In this paper, we propose a method for the automatic detection of patient’s emotions in healthcare data using supervised machine learning approaches. For this purpose, we created a new dataset named EmoHD, comprising of 4,202 text samples against eight disease classes and six emotion classes, gathered from different online resources. We used six different supervised machine learning models based on different feature engineering techniques. We also performed a detailed comparison of the chosen six machine learning algorithms using different feature vectors on our dataset. We achieved the highest 87% accuracy using MultiLayer Perceptron as compared to other state of the art models. Moreover, we use the emotional guidance scale to show that there is a link between negative emotion and psychological health issues. Our proposed work will be helpful to automatically detect a patient’s emotion during disease and to avoid extreme acts like suicide, mental disorders, or psychological health issues. The implementation details are made publicly available at the given link: https://bit.ly/2NQeGET.

## Introduction

Emotions indicate the way of communication and play a significant role in daily life applications *e.g.*, general health of the public, crisis reaction, and feedback analysis (Hasan, Rundensteiner & Agu, 2019). In human beings, emotions can be exchanged through feelings, text, and speech. Recognition of emotions and feelings is comfortably comprehended between humans but it is not possible for a computer to realize the type of emotion and its intensity. The field of research shows much interest in emotion analysis as it provides a path in human-machine communication.

There has been a lot of research done in the field of emotion detection using textual data, but the applications are diverse. The literature shows that the text is the fundamental source of emotion detection because there is a variety of textual data covering social media content (Facebook and Twitter, *etc.*), blogs, news, forums, chats, and discussion communities data. Moreover, the prediction of emotions related to health data can be very beneficial to detect positive and negative emotions because negative emotions can be harmful and lead to some dangerous state either mental or physical. Poor emotional health affects human health in terms of causing depression, anxiety, loneliness, mental breakdown, and similar problems. Therefore, there is a need to perform a study on such a type of problem where emotion from text is detected and mapped to the domain of Psychology.

There is no agreement in the writing on a particular meaning of feeling or emotion. A “feeling” is regularly underestimated in itself and frequently is characterized concerning a rundown of descriptors, for example, outrage, nauseate, joy and trouble, and so on. Feelings can change regularly and rapidly in a brief time frame period during diseases. These are inside states that we see or express (*e.g.*, through voice or motion); however, they are not intelligent and significant. On the other side, behaviors incorporate profoundly complex elements, data from express and certain perspectives are shown throughout longer time scales. For example, “happiness or joy”, as one of the enthusiastic states is achieved by commonly good emotions. When a man is in good health he feels happy while during any disease human emotion normally becomes negative. During any disease, the human feels sad, unpleasant, and sometimes feel disappointed if the disease remains for a long time and such emotions can lead to committing suicide in severe cases. In our work, we extracted the emotions of patients from different disease news available on different online platforms.

Traditional techniques to recognize the emotion in text are comprised of four standard methods (Canales & Martínez-Barco, 2014; Azam, Tahir & Mehmood, 2020b): (1) Keyword Based Method (2) Lexicon Based Method (3) Machine Learning Based Method and (4) Hybrid Based Method. The conventional and easy method is the first one, *i.e.,* Keyword Based Method. In this method, the emotional keyword is found out in the input sentence and some matching pattern is followed to extract the specific keyword (Binali, Wu & Potdar, 2010). Natural Language Processing (NLP) techniques can be applied for tokenizing the input text and some analysis is performed to find the intensity of words as well. Upcoming to the Lexicon Based Method, it makes use of emotion lexicons to find emotion in a sample of text (Joshi et al., 2016). This method finds a keyword on the basis of probability the sense to take positive or negative. A survey paper (Azam, Tahir & Mehmood, 2020b) shows that there are some emotion lexicons that are publicly available. Next comes the Machine learning methods consisting of two types, *i.e.,* Supervised and Unsupervised. Classification and Clustering techniques are used for these two types, respectively (Pang et al., 2019; Asghar et al., 2019; Fiorini et al., 2020; Raju & Mani, 0000). Finally, the last method is the Hybrid method that works on combining two or more above mentioned techniques for emotion classification in text documents (Chen & Hao, 2019).

In this paper, we perform emotion classification on health-related text data. For this purpose, we target the prevailing diseases of Pakistan. We use a publicly open API that is known as Parallel Dots to label the text data related to each of the different diseases. This labeled data is then trained on six different supervised machine learning classifiers. Feature engineering is applied on the basis of Count Vectorization and Term Frequency-Inverse Document Frequency (TF-IDF). The performance comparison of each classifier is analyzed and the best performing model is tested on the sample dataset. After the prediction of emotion from the text, we filter the negative emotions and map them to health issues. The mapping is done with a reference to an emotional guidance scale.

The major contributions of this research work are stated as follow:

• We present the first comprehensive work on detection or recognition of patient’s emotions of seven major diseases of Pakistan through text obtained from different online health sources and social sites.
• We also propose an extensive dataset, named EmoHD, that comprises 4202 text samples related to major diseases. Our dataset also has an emotion label from 6 different emotions along with each text sample.
• We performed a detailed analysis of six different machine learning algorithms based on different feature engineering techniques on the proposed dataset. MultiLayer Perceptron model using count vector features outperforms in results with 87% accuracy than all other 5 models.
• We also filter out the negative emotions and map them to the Psychological health issues to produce an alert so that any mishap can be avoided.

The rest of the paper is organized as follows: First, we present the related work in ‘Related Work’. Next, the dataset created for this research is presented in ‘Dataset’. Furthermore, we discuss the proposed methodology in ‘Methodology’. In ‘Experiments and Results’, we provide the results obtained from our experiments. Additionally, we provide the use cases of the proposed method and conclusion in ‘Use Cases’ and ‘Conclusion’ respectively. Finally, the future directions are given in the ‘Future Directions’.

## Related Work

By the time Artificial Intelligence (AI) was favored in the field of Computer Science (CS), this domain came up essentially with amazing solutions to many problems including Natural Language Processing (NLP) and linguistics strategies (Acheampong, Wenyu & Nunoo-Mensah, 2020). Different classification techniques are now trading in CS regarding sentiments and emotions from textual data. Moreover, the researchers utilized the insights of emotion classification from Twitter data to predict election results using the lexicon-based approach (Srinivasan et al., 2019). A corpus of tweets related to Hillary Clinton and Donald Trump have been used as a dataset in their methodology. They were able to correctly predict the swing directions of 17 out of 19 states. Similarly, another study based on the Twitter data for emotion classification uses the Circumplex model of affect to find emotion in tweets (Hasan, Rundensteiner & Agu, 2019). They use Naive Bayes, Support Vector Machine, and Decision Tree in their study.

In Rahman, Islam & Ahmed (2017), the authors detect emotions not only from the text but also from emoticons. Their work is based on keyword analysis, keyword negation analysis, a set of proverbs, emoticons, short form of words, and exclamatory words. They calculate the success rate and achieve 87% score for their technique. There are also some studies available in the literature that make use of NLP techniques by defining manual dictionaries of basic expressions (Gosai, Gohil & Jayswal, 2018). NLP techniques used by Gosai, Gohil & Jayswal (2018) include stop words removal, POS tagging, tokenization, lemmatization etc. In the field of research, the emotion analysis from text is performed by multiple studies. As in Chatterjee et al. (2019), the authors have used Deep Learning (DL) and Big Data for understanding emotions in text. A further study (Asghar et al., 2019), analyzes the performance of different machine learning algorithms to efficiently detect emotions from online content. Additionally, a comparative study has been made by Mohsen, Idrees & Hassan (2019) to analyze emotion from the text for opinion mining.

A paper (Azam, Tahir & Mehmood, 2020b), presents a survey on available resources for emotion analysis including the datasets, lexicons, and approaches. Based on their study, it can be said that emotion labeled datasets are not in large quantity and not publicly available. For this purpose, there are many research studies of emotion classification and detection from the text that seek help from some third-party API, *i.e.,* Parallel Dots (Komprehend, 2020). Parallel Dots have models that are trained on a huge amount of documents. The common use cases of the API include sentiment and emotion analysis. It can also be used for intent detection and abusive text recognition. Making use of this API, the Whatsapp chats as textual data have been used for text classification for behavioral analysis (Dahiya, Mohta & Jain, 2020). In the studies (Masterton Perry, Nurmikko-Fuller & Nunes, 2019; Bose et al., 2019), twitter data is used for the evaluation of sentiment analysis using Parallel Dots. Besides, there are much other research works (Anderson, 2019; Marerngsit & Thammaboosadee, 2020; Rajalakshmi & Rajagopalan, 2019) that apply Parallel Dots for detecting emotions from text documents. Li, Baucom & Georgiou (2020) proposed a network in which they have shown that emotions are directly linked to human behavior. This paper shows that for the recognition of human behaviour, emotion-related information plays an important role. Emotion recognition also plays an important role to detect fake news. Reyes-Menendez, Saura & Filipe (2019) has led a precise survey of the writing on fake news. Their results show that current state of the art methods largely rely on emotional approaches to detect fake news.

Prediction of emotion in the text written by a disease suffering person or any patient shows the presence of more negative feelings as compared to positive ones. High levels of fear, anxiety, or anger and similar type of emotions or notions subsist in patients (Mazzocco et al., 2019). A lot of research papers are found describing the presence of negative emotions in patients (Derry, Reid & Prigerson, 2019; Annunziata et al., 2019; Zare et al., 2019). A patient suffers from physical, emotional, and psychological challenges linked with their daily life (De Los Rios et al., 2020). The existence of negative emotion by the end leads the way to depression, mental illness, and health issues (Young, Sandman & Craske, 2019). Additionally, research proves that stress and unbalance emotions have a greater bias towards depression (Maydych, 2019).

## Dataset

In this section, we discuss about the dataset used for emotion detection in patients from the text. For automatic recognition of emotions in patients through text, as per our knowledge, no comprehensive public dataset is available. In this research work, we propose a new benchmark dataset for automatic recognition of emotions in healthcare text data and we name our dataset as EmoHD. In recent years datasets have been considered an important part of extracting useful information. A dataset like fer2013 (Nicole, 2019) is instrumental in the detection or recognition of facial expressions.

However, for the purpose of automatic recognition of emotion in patients through healthcare data or from news relevant to diseases is basic for progressing research in this field. We contend that a comprehensive healthcare news data should have the following four properties: (1) a large number of samples along with disease name label, (2) a large number of samples per class, (3) appropriate value of every emotion along with text, (4) and lastly, appropriate emotion label against each text which makes a way to recognize or detect real-time emotion of patient. To detect emotion in a patient’s text data suffering from a disease, we need the emotion labeled dataset. However, due to the non-existence of such a dataset, there is a dire need to build a dataset that is emotionally labeled distributed over different diseases.

### Labelling of EmoHD dataset

#### Data collection

To build the required dataset, we collect English text from different online sources including Health News sites *e.g.*, Medisys (Rortais et al., 2010) and Systematized Nomenclature of Medicine-Clinical Terms (SNOMED-CT) (Donnelly, 2006), Discussion forums & Communities. Further, we also collect some text samples from Google News (http://news.google.com/) related to the relevant disease by manually searching with disease keywords. Further details about dataset collection can be seen in our previous paper (Azam, Tahir & Mehmood, 2020a). We crawled the dataset from different online sources and save the text into an excel file. There are currently a total of 4,202 text samples in our dataset related to different prominent diseases and we are also working to expand it further.

#### Disease classes

For this purpose, first of all, we target the dominant diseases of Pakistan. According to the Seasonal Awareness and Alert Letter (SAAL) (National Institute of Health, Government of Pakistan, 2020) of October 2019–April 2020 issued by the Ministry of National Health Services, Regulations & Coordination, Government of Pakistan, National Institute of Health (NIH) Islamabad Pakistan, Measles, Dengue, Typhoid, Malaria, Acute Hepatitis, HIV, Ebola, Cancer, and some others are the dominant diseases of Pakistan. Moreover, we also include the disease which is trending in the world 2020, *i.e.,* Coronavirus or COVID-19. Therefore, we prepare our own emotionally labeled dataset allocated on the chosen diseases. There are a total of eight disease classes in the EmoHD dataset which are HIV/AIDS, Dengue, Hepatitis, Malaria, Influenza, Coronavirus, Cancer, and others respectively. Other class means if text sample is not related to the mentioned seven classes then we gave that text to another class label. We can say in other class, text can be related to any other disease but not from these seven mentioned disease classes. In this way, at the point of data collection, we already know the disease class of that text sample as we are fetching the picked out disease type data from online resources.

#### Emotion class labelling

The Emotion label is given to the text sample by using Parallel Dots API (Komprehend, 2020). It is an Artificial Intelligence (AI) platform created using advanced Deep Learning (DL) and Machine Learning (ML) techniques. This API labels the text into 6 classes as follows: (1) Happy (2) Sad (3) Angry (4) Excited (5) Bored (6) Fear. To use this API, we need an API_Key and it returns a JSON response having a confidence score for each of the emotion labels (Happy, Sad, Angry, Excited, Bored, or Fear). After getting the probability of every six emotions from the Parallel Dots API, we then assign the final emotion label to every text on the basis of the higher probability of emotion class. So now in our excel file, we have text along with disease label and final label of emotion against each text. Also, we have the probability of every emotion class against each text. In this way, we labeled the dataset containing 4202 samples.

#### Dataset splits

We randomly split our dataset into the training and testing with the ratio of 80% and 20%. After splitting our dataset into the training and testing part, there are a total of 3362 samples in training dataset and 840 total samples in the test dataset.

### Properties of EmoHD

#### Text specialities of EmoHD dataset

We did an analysis on our collected dataset and it can be seen in the Table 1 that there are a total of 1634319 number of words in our dataset and if we talk about unique number of words then it is around one lac that is 91988. Similarly. the total number of characters, number of numerics, unique Bigrams, and Trigrams can also be seen in Table 1.

**Table 1:** Overall statistics of different features of the EmoHD dataset.

**Data features**		
**Sr#**	**Feature**	**Count**
1	**No. of characters**	12090922
2	**No. of words**	1634319
3	**No. of numerics**	64543
4	**Unique unigrams**	91988
5	**Unique bigrams**	827475
6	**Unique trigrams**	1159467

#### Emotion class wise distribution of EmoHD dataset

Table 2 shows the emotion class-wise distribution for each text sample. It is evident in Table 2 that the Angry emotion class has a high number of text samples that are 1,343 and the Bored emotion class has the least number of text samples that are only 22. Sad emotion class has also a significantly less number of samples than other emotion classes. Since, we are talking about emotions of patients during a specific disease then it is obvious that if someone has a disease then there will be less chance that the person will feel bore during that disease. It is natural that is why our dataset has less number of bored emotion class samples. Moreover, It can be seen from the given Table 2 that the class imbalance problem is present in our dataset. This is because of text we got against each disease and we can overcome on class imbalance problem by applying oversampling or under-sampling techniques.

**Table 2:** Overall distribution of EmoHD dataset with respect to the emotion class label.

**Data statistics**		
**Sr#**	**Emotion class**	**Count**
1	**Angry**	1343
2	**Excited**	1215
3	**Fear**	742
4	**Happy**	522
5	**Sad**	358
6	**Bored**	22
	**Total**	**4202**

#### Disease class wise distribution of EmoHD dataset

**Figure 1:**
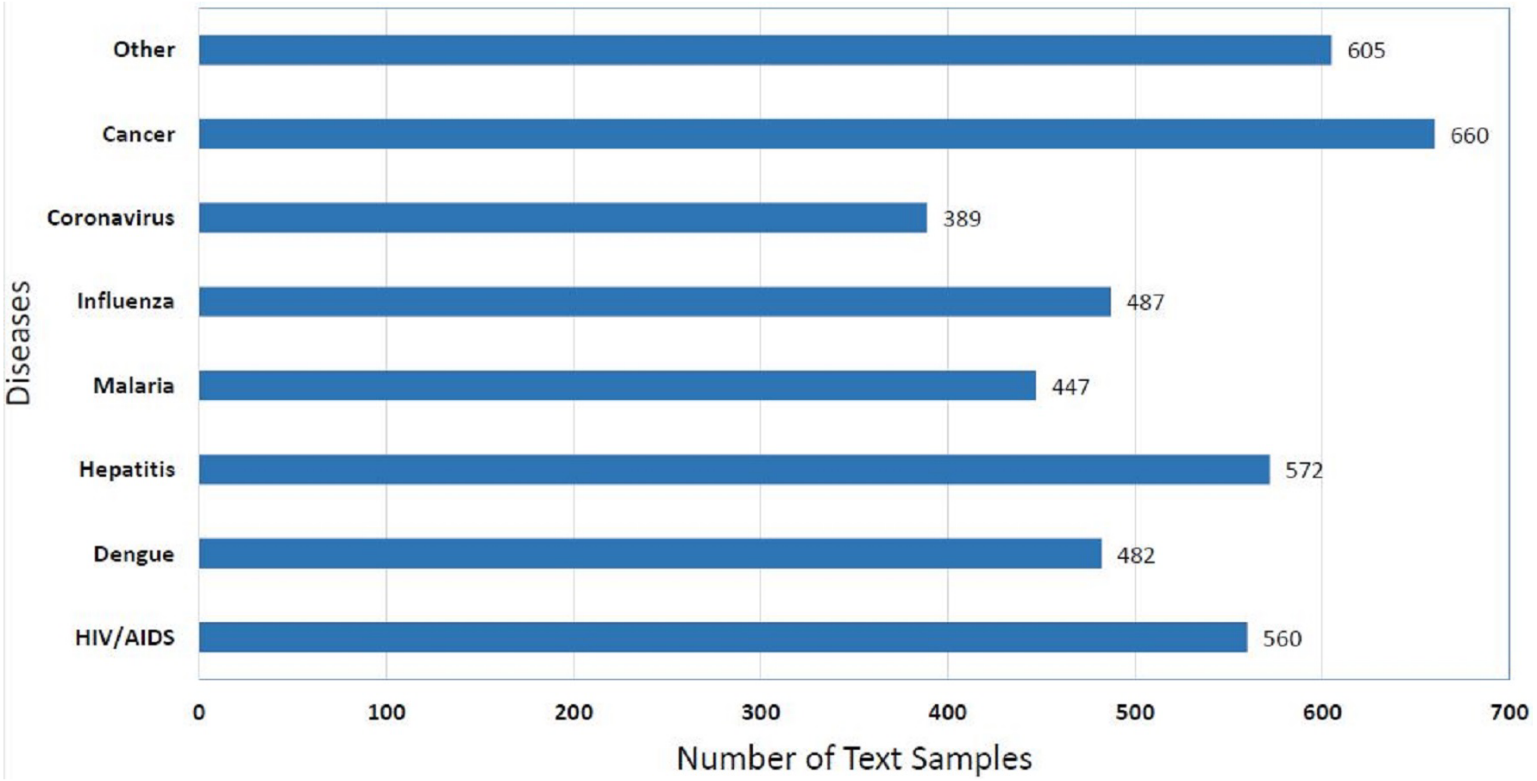
Distribution of the EmoHD dataset with respect to disease class label.

Statistics of distribution of disease class of EmoHD dataset can be seen in the Fig. 1. It can be seen in given Fig. 1 that the Cancer class has a high number of samples that are 660. It is evident from the Fig. 1 that the class imbalance problem does not explicitly exist in disease class wise distribution of the EmoHD dataset but we have seen in Table 2 that when we see emotion class wise distribution of these samples then class imbalance balance problem is present there as we have discussed the reason in the previous section.

## Methodology

**Figure 2:**
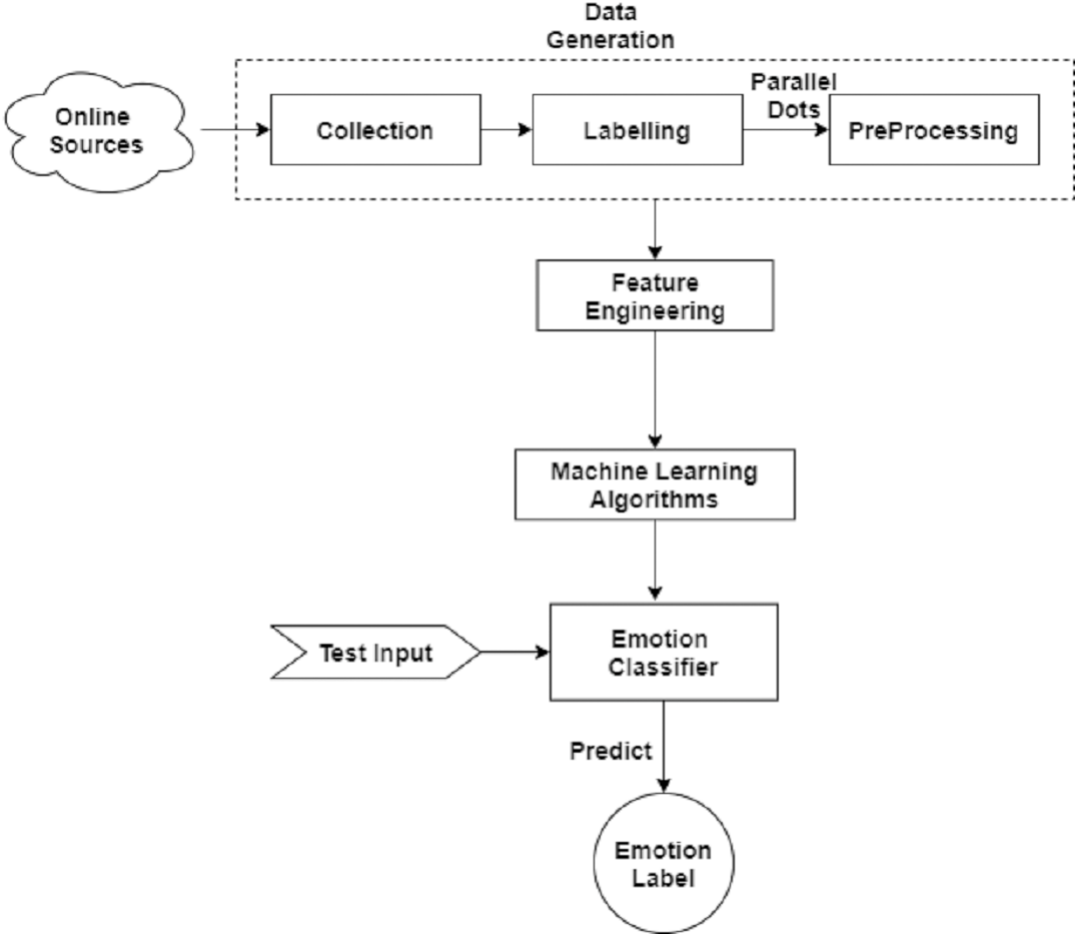
Basic flow diagram of predicting emotion in patients during specific disease with the help of online available disease related news.

In this section, we describe the methodology adopted for emotion detection in patients from text data. The architecture diagram of the proposed methodology is given in the Fig. 2. First, we describe the pre-processing of the dataset in detail then data re-sampling is applied. Next, we perform feature engineering on the data to train the model.

### Pre-processing

Our first step is the cleaning of the data in order to obtain better results. We can achieve stronger results by doing some basic pre-processing steps including lowercase conversion, punctuation removal, stop words removal, etc. Each of the text cleaning steps is mentioned below.

#### Lowercase conversion

The first step of pre-processing is the lower case conversion. For this purpose, we clean the data by transforming the text into the lower case to avoid having multiple copies of the same words. For instance, ‘Happy’ and ‘happy’ must be considered as a single word but due to case differences they are counted as different words. Therefore, we apply the lower case conversion technique to avoid such ambiguities.

#### Punctuation removal

The next step is to remove punctuation from the text, as it does not add any useful information while training the model. Removing all punctuation occurrences from text data will help the model to learn functional features instead of non-functional instances.

#### Removal of stop words

Stop words should be removed from the text data as they do not add much meaning to the machine learning model. For this purpose, we use a predefined library of python, *i.e.,* nltk.corpus (Pythonspot, 2016) for English stop words.

#### Common words removal

Common words are removed from the textual data because of the high similarity in the vocabulary. The overlap in vocabulary affects the evaluation scores during the training of machine learning classifiers. These words are removed as their presence will not be of any use in the classification of our text data.

#### Rare words removal

Similarly, just as we removed the most common words, we also remove rarely occurring words from the text. They are so rare, hence, the association between them and other words is dominated by noise.

#### ReSampling

It can be seen in the Table 2 that the emotion class distribution is not evenly distributed and it clearly shows the unbalanced data. To handle unbalanced data, we need to perform data re-sampling. Re-sampling technique (Tian et al., 2019) can either be Over-Sampling or Under-Sampling. We try both of these approaches and resample the data based on the majority and minority class respectively.

Moreover, after balancing the data, the analysis is performed based on two perspectives, *i.e.,* including minority class as well as excluding minority class.

### Feature engineering

After getting clean text, the next step is to perform feature engineering using the standard methods. In this phase, we transform the raw text data into feature vectors. This is implemented using different approaches including Count Vectorization and Term Frequency-Inverse Document Frequency (TF-IDF) to obtain relevant features.

#### Count vectorization

In Natural Language Processing (NLP) toolbox, Count Vectorization has an important role. The number of times a word or term appears in the documents are referred to as count vectors and that is why Count vectorizer is also termed as Term Frequency vectorizer (Sekhar et al., 2020). In count vectorizer, we do not simply catch the presence of words for guaranteed report yet additionally catch how frequently it happens. Consequently, the text data is presented as a vector of words alongside the occasions of the terms that happen in that text sample.

#### Term frequency-inverse document frequency

Realizing exactly when a word appears in text data and when they show up consistently or inconsistently across the dataset isn’t extremely useful. Regardless of whether a word shows up habitually in certain documents yet less oftentimes in others can be truly valuable (Bounabi, El Moutaouakil & Satori, 2019). Therefore, TF-IDF comes in there and plays its vital role. TF-IDF score is composed of two terms as mentioned below:

\begin{eqnarray*}TF(w)= \frac{\text{Number of times word (w) appears in a document}}{\text{Total number of words in the document}} \end{eqnarray*}

\begin{eqnarray*}IDF(w)=\text{log}( \frac{\text{Total number of documents}}{\text{Number of documents with word (w)}} ) \end{eqnarray*}

Further, TF-IDF vectors can be generated at different levels of input tokens, *i.e.,* Words, Characters, and N-grams (Bansal, 2019). The Word Level TF-IDF is a matrix that represents TF-IDF scores of every word in different documents or archives. Similarly, N-gram Level TF-IDF is actually the combination of N terms together and the resultant matrix describes the tf-idf scores of N-grams. On the other hand, the CharLevel TF-IDF score is based on the characters present in the corpus.

### Machine learning models

In text classification, the next step is to train a machine learning model or classifier using the selected features as described in ‘Feature Engineering’. We used six different supervised machine learning models and have done a detailed comparative analysis of the results of these models. There are many papers that make use of these six ML models to classify the dataset (Asghar et al., 2019). There are some survey papers that discuss the approaches and techniques (Hakak et al., 2017; Alswaidan & Menai, 2020). Therefore, we target those state of the art models. Models we used for training and testing are described as follows:

#### Multinomial naive bayes (MNB)

MNB is specially designed for text data and it is a particular version of Naive Bayse (Balakrishnan & Kaur, 2019). Multinomial Naive Bayes classifier utilizes multinomial dissemination for every one of the features. MNB just tells us that each probability of feature given class is a multinomial dispersion instead of some other dissemination. This functions admirably for information that can undoubtedly be transformed into counts, for example, the word includes in content.

#### MultiLayer perceptron (MLP)

**Figure 3:**
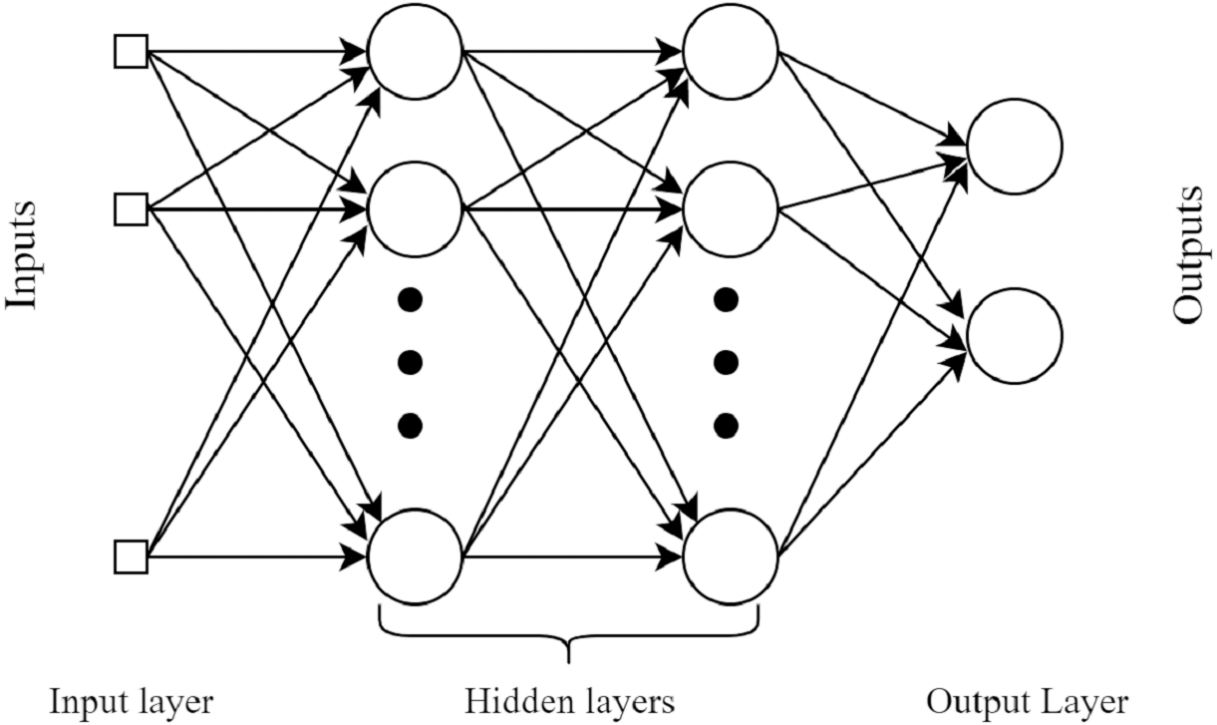
A basic four layers example architecture of MultiLayer Perceptron.

MLP is a feed-forward neural network (FFNN). This classifier uses the backpropagation method during the learning of data (Labani et al., 2018). A MLP comprises of at least 3 layers: an input layer, a hidden layer, and a yield layer. Aside from the input layer, every node in every layer is a neuron that utilizes a nonlinear activation function. The basic architecture diagram of MLP is shown in Fig. 3.

#### Logistic regression (LR)

Logistic regression looks like a regression algorithm by name but actually, it is a classification algorithm. Logistic regression uses the sigmoid function. The sigmoid function can be defined as given in Eq. (1).

(1) \begin{eqnarray*}g(x)= \frac{1}{1+{e}^{{-}^{x}}} \end{eqnarray*}

while x in Eq. (1) can be any number or it can be any formula. It is a linear classifier that calculates the probabilities using logistic function to find the relation between categorical dependent and independent variables (Genkin, Lewis & Madigan, 2007; Shah et al., 2020).

#### Support vector machine (SVM)

SVM is a supervised ML algorithm (Tong & Koller, 2001). The model is very simple and many people preferred to use this model because of less computational power and it gives significant accuracy. SVM not only performs linear classification but also performs non-linear classification very well with the help of kernel trick. The basic idea of this model is very simple, the algorithm draws a hyper-plane or line that divides the data into categories or classes. Hinge loss is used to draw a line that helps in maximizing the margin between line and data points. For an expected yield t = ±1 and a classifier score z, hinge loss can be defined as given in Eq. (2).

(2) \begin{eqnarray*}Los{{s{}_{H}{}_{i}{}_{n}}_{g}}_{e}=max(0,1-t\ast z)\end{eqnarray*}

It can be used for both regression problems and classification problems but mostly it is used for classification problems. This model is very helpful for text categorization. That’s why we used this model to predict emotion class from given text samples of the EmoHD dataset. It performs very well when data has high dimensions but its efficiency compromises with noisy data.

#### Random forest (RF)

Random Forest is an administered learning calculation that is utilized for both classification and regression (Onan, Korukoğlu & Bulut, 2016). Nonetheless, it is predominantly used for classification. As we all know, timberland is made up of trees, and more trees imply a more vigorous woodland. Essentially, arbitrary woodland calculation makes choice trees on information tests and afterward gets the expectation from every one of them lastly chooses the best arrangement by methods for casting a ballot. It is an outfit technique that is superior to a solitary choice tree since it diminishes the over-fitting. Random forest calculation runs productively in enormous information bases and creates exceptionally precise forecasts by assessing missing information.

#### eXtreme gradient boosting (XGB)

XGB uses a gradient boosting framework and basically, it is an improved version of gradient boosting algorithm (Ria et al., 2020). It is a more regularized model that uses L1 and L2 regularization which helps to improve model generalization ability, thus overcoming the overfitting problem. This model should not be used for image recognition or if there are few training samples than number of features.

### Evaluation metrics

To evaluate the performance of each mentioned classifier, standard evaluation metrics have been used, including Accuracy, Macro Precision, Macro Recall, and F1 score.

The most intuitive metric to evaluate a machine learning model is “Accuracy” and it is simply the ratio of the number of correctly predicted documents and the total number of documents in the dataset. While the ratio of correct predictions to the total number of positive predicted documents is known as “Precision”. A high score of precision shows a low false positive rate that is a good side of the model. In contrast, Recall is the sensitivity of a classifier that is the ratio between positive predictions to all predictions in actual class. Sometimes, Precision and Recall are not enough to evaluate the model so F1-score is calculated which is the weighted average of Precision and Recall. Because it gives importance to both false positives and false negatives. Also, F1 is far better than accuracy if the uneven distribution of classes exists in the dataset. The mathematical expression for each of the metrics is noted below.

(3) \begin{eqnarray*}Accuracy= \frac{\text{TP +TN}}{\text{Total}} \end{eqnarray*}

(4) \begin{eqnarray*}Precision= \frac{\text{TP}}{\text{TP + FP}} \end{eqnarray*}

(5) \begin{eqnarray*}Recall= \frac{\text{TP}}{\text{TP + FN}} \end{eqnarray*}

(6) \begin{eqnarray*}F1=2\ast \frac{\text{(Recall * Precision)}}{\text{(Recall + Precision)}} \end{eqnarray*}

where, TP = True Positives, TN = True Negatives, FP = False Positives, FN = False Negatives, Total = P + N, P = No. of positive samples, N = No.of negative samples.

## Experiments and Results

We compare the results of six machine learning models on the proposed EmoHD dataset. In this section, first, we present the performance comparison of different classifiers using a variety of features. Next, we describe test results achieved from test data samples. Finally, we analyze the presiding emotions in the test dataset.

### Results & analysis

We trained all six machine learning algorithms as described in ‘Machine Learning Models’ and check the results of every model on our proposed EmoHD dataset. We evaluated these models using accuracy. Quantitative analysis of results of each model are described below. Overall accuracy of MNB model on proposed EmoHD dataset is 74% using character level tf-idf vectors. Overall accuracy of MLP model on proposed EmoHD dataset is 85% using character level tf-idf vectors and maximum F1 score we obtained is 94%. Overall accuracy of LR model on proposed EmoHD dataset is 85% using character level tf-idf vectors and maximum F1 score we obtained is 94%. Overall accuracy of SVM model on proposed EmoHD dataset is 85% using character level tf-idf vectors and maximum F1 score we obtained is 95%. Overall accuracy of RF model on proposed EmoHD dataset is 87% using character level tf-idf vectors and maximum F1 score we obtained is 96%. Overall accuracy of XGB model on proposed EmoHD dataset is 84% using character level tf-idf vectors and maximum F1 score we obtained is 94%.

### Ablation study

We trained six machine learning models using different features including Count Vectors and TF-IDF feature vectors. This is done by adopting two approaches, *i.e.,* Including the minority class and excluding the minority class. Based on these features, we evaluate the performance of those six models and the scores for each evaluation metrics are given in Table 3. This Table 3 shows the scores when we include the minority class, *i.e.,* the “Bored” class. It can be seen that we achieve very satisfactory results for each of the classifiers. In the case of Count Vectors, MLP is at the top position by giving 87% accuracy score. With WordLevel TF-IDF features, MLP and RF are giving the highest accuracy of 86%. Using the N-gram vectors and CharLevel TF-IDF, again RF is providing a high accuracy that is 86% respectively. Contrary to this, Table 4 represents scores for each classifier along with different features when excluding the minority class. Here, RF is giving outstanding results with Count Vectors and CharLevel TF-IDF features with 86% accuracy. While in this scenario, XGB along with RF is showing high scores of accuracy for WordLevel and N-Gram vectors with 84% accuracy.

**Table 3:** Comparison and analysis of quantitative results of six different machine learning models using different four feature while including minority emotion class.

**Sr#**	**Model**	**Feature**	**Accuracy**	**Macro precision**	**Macro recall**	**Macro F1**
**1**	MNB	Count vectors	72%	0.73	0.72	0.72
		WordLevel TF-IDF	63%	0.63	0.62	0.62
		N-Gram vectors TF-IDF	60%	0.61	0.60	0.60
		CharLevel vectors TF-IDF	54%	0.55	0.55	0.55
**2**	MLP	Count vectors	87%	0.87	0.87	0.87
		WordLevel TF-IDF	86%	0.86	0.86	0.86
		N-Gram vectors TF-IDF	85%	0.85	0.85	0.85
		CharLevel vectors TF-IDF	83%	0.83	0.83	0.83
**3**	LR	Count vectors	86%	0.86	0.86	0.86
		WordLevel TF-IDF	75%	0.75	0.75	0.75
		N-Gram vectors TF-IDF	74%	0.74	0.74	0.74
		CharLevel vectors TF-IDF	59%	0.60	0.60	0.60
**4**	SVM	Count vectors	76%	0.76	0.77	0.76
		WordLevel TF-IDF	83%	0.83	0.83	0.83
		N-Gram vectors TF-IDF	82%	0.82	0.83	0.82
		CharLevel vectors TF-IDF	62%	0.63	0.64	0.63
**5**	RF	Count vectors	86%	0.86	0.87	0.86
		WordLevel TF-IDF	86%	0.86	0.86	0.86
		N-Gram vectors TF-IDF	86%	0.86	0.86	0.86
		CharLevel vectors TF-IDF	86%	0.86	0.87	0.86
**6**	XGB	Count vectors	83%	0.84	0.83	0.83
		WordLevel TF-IDF	85%	0.85	0.85	0.85
		N-Gram vectors TF-IDF	84%	0.85	0.84	0.85
		CharLevel vectors TF-IDF	85%	0.86	0.86	0.86

**Table 4:** Comparison and analysis of quantitative results of six different machine learning models using different four feature while excluding minority emotion class.

**Sr#**	**Model**	**Feature**	**Accuracy**	**Macro precision**	**Macro recall**	**Macro F1**
**1**	MNB	Count vectors	67%	0.67	0.66	0.66
		WordLevel TF-IDF	55%	0.55	0.54	0.54
		N-Gram vectors TF-IDF	54%	0.54	0.53	0.53
		CharLevel vectors TF-IDF	46%	0.47	0.46	0.46
2	MLP	Count vectors	83%	0.83	0.83	0.82
		WordLevel TF-IDF	83%	0.82	0.82	0.82
		N-Gram vectors TF-IDF	83%	0.83	0.82	0.82
		CharLevel vectors TF-IDF	83%	0.83	0.82	0.82
**3**	LR	Count vectors	82%	0.82	0.81	0.81
		WordLevel TF-IDF	72%	0.72	0.71	0.71
		N-Gram vectors TF-IDF	72%	0.72	0.71	0.71
		CharLevel vectors TF-IDF	52%	0.52	0.51	0.51
**4**	SVM	Count vectors	74%	0.74	0.74	0.74
		WordLevel TF-IDF	81%	0.81	0.81	0.81
		N-Gram vectors TF-IDF	80%	0.80	0.80	0.80
		CharLevel vectors TF-IDF	57%	0.57	0.58	0.57
**5**	RF	Count vectors	86%	0.86	0.86	0.86
		WordLevel TF-IDF	84%	0.84	0.84	0.84
		N-Gram vectors TF-IDF	84%	0.84	0.84	0.84
		CharLevel vectors TF-IDF	86%	0.86	0.86	0.86
**6**	XGB	Count vectors	84%	0.84	0.83	0.83
		WordLevel TF-IDF	84%	0.84	0.84	0.84
		N-Gram vectors TF-IDF	84%	0.84	0.84	0.84
		CharLevel vectors TF-IDF	85%	0.85	0.85	0.85

The very first limitation of the methodology is the selection of a sample data. As we have used the basic emotion labels for data labelling, we find that there were very less samples found for the Bored class. Due to the existence of minority class, the performance of the models suffer. Another hurdle is that there were no participants available who can do the manual labelling of such huge dataset. We had not the ability to find the geographic scope of the participants who can help us in data labelling. Therefore, we preferred to utilize the online resource, *i.e.,* Parallel dots API. It is known that the larger the sample, the more precise your results will be. If your sample size is too small, it will be difficult to identify significant relationships from the data. Therefore, we somehow managed to create a reasonable amount of data samples to initiate the study. Hence, we plan to increase the data in future. Depending on the scope of our research topic, although there are many prior research studies available that are relevant but there were very few who have used the online health data and extract the emotions. Our study is not limited to extraction of emotion but also to the mapping of different psychological health issues. When there is very little or no prior research on a specific topic, you may need to develop an entirely new research typology. In this case, discovering a limitation can be considered an important opportunity to identify new gaps in the prior literature and to present the need for further development in the area of study.

### Test data analysis

Choosing the best performing classifier and testing on the new and unseen text sample is analyzed in this section. For this purpose, we collect 10 text data samples from different online health resources for each five emotion classes (excluding the minority class). Then, we predict the label of text data with the help of the best performing trained model. Out of the total 50 test samples, the total correctly predicted labels are 41 and we achieve 82% accuracy on the test data. Table 5 shows the correctly predicted samples with their counts.

**Table 5:** Test data results.

**Emotion**	**Actual label ount**	**Truly predicted count**
**Angry**	10	8
**Excited**	10	7
**Happy**	10	8
**Fear**	10	9
**Sad**	10	9
**Total**	**50**	**41**

### Models computational analysis

An extensive analysis of training and testing time of models used to predict emotion in healthcare data is also performed. Results of training and testing time of models can be seen in Table 6. It is clear from the results that the MLP model has the highest 563 s training time while the MNB model has a minimum of 0.01 s training time than all the other models. We have seen in Table 3 that MLP has the highest accuracy and the testing time of this model is 0.04 s. It is also observed that using character level vectors features takes more training time in all models except MLP as compared to using Count Vectors, Word Level tf-idf, and N-Gram Vectors features.

**Table 6:** Training and testing time of models in seconds.

**Model**	**Feature**	**Training time**	**Testing time**
**MNB**	Count vectors	0.10	0.04
	WordLevel TF-IDF	0.05	0.007
	N-Gram vectors	0.01	0.000
	CharLevel vectors	0.10	0.02
**MLP**	Count vectors	563.3	0.04
	WordLevel TF-IDF	500.1	0.03
	N-Gram vectors	454.2	0.02
	CharLevel vectors	495.1	0.08
**LR**	Count vectors	10.6	0.007
	WordLevel TF-IDF	1.92	0.002
	N-Gram vectors	1.98	0.001
	CharLevel vectors	8.99	0.01
**SVM**	Count Vectors	63.0	13.1
	WordLevel TF-IDF	48.8	9.2
	N-Gram vectors	49.8	9.4
	CharLevel vectors	397.5	77.8
**RF**	Count vectors	16.0	0.23
	WordLevel TF-IDF	9.62	0.09
	N-Gram vectors	10.2	0.10
	CharLevel vectors	32.2	0.35
**XGB**	Count vectors	27.7	0.25
	WordLevel TF-IDF	43.7	0.10
	N-Gram vectors	10.2	0.12
	CharLevel vectors	337.9	0.59

### Mapping of negative emotions

In this section, we map the extracted emotions to the domain of Psychology to predict the feeling of a disease suffering person. As discussed in the ‘Related Work’ that according to the Research of Psychology, there exists a relationship between disease suffering person and negative emotions. These negative emotions eventually lead to the extreme stage like suicide or any other mental disorder. The mapping of negative emotion can be achieved by seeking help from the book entitled as “Ask and It is Given” (Hicks & Hicks, 2004).

**Figure 4:**
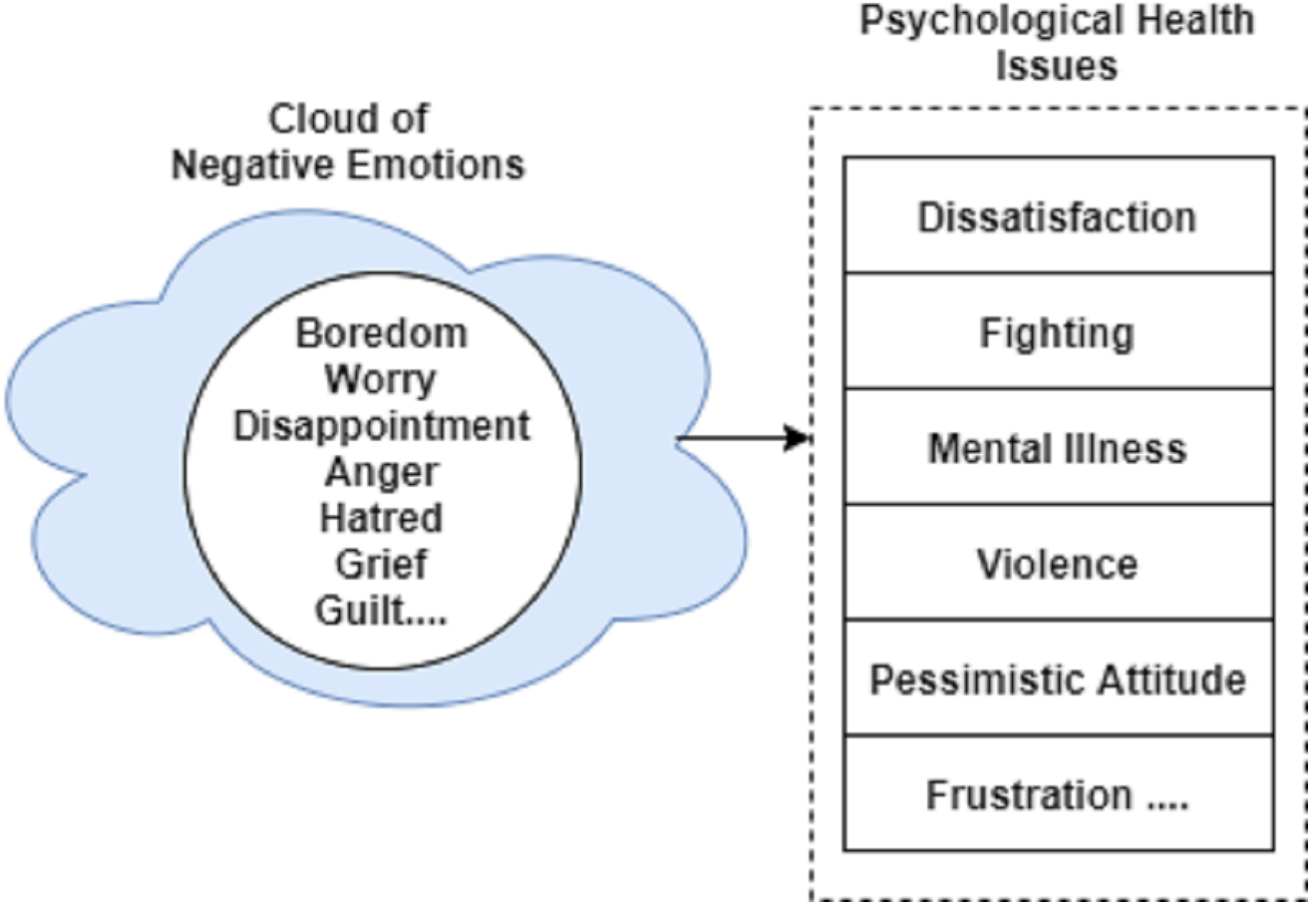
Negative emotions leading to psychological health issues.

In the book (Hicks & Hicks, 2004), the Emotional guidance scale is available that is a spiral model of emotions and feelings. An upward spiral shows all positive emotions while a downward spiral shows the negative emotions with the increasing intensity in the downward direction. We use a downward spiral as it contains negative emotions. Negative emotions like bored, fear, anger, *etc.*, lead to the last stage of negativity, *i.e.,* depression, powerlessness, and insecurity (Yıldız & Solakoglu, 2019; Shapero et al., 2019). Figure 4 shows a sample of a cloud of negative emotions that eventually leads to psychological health issues including dissatisfaction, mental disorder, and violence, etc.

## Use Cases

We believe that our proposed methodology will help the companies, organizations, and online communities to respond to patient’s needs and struggles. By integrating our built methodology, a web-based system can be designed to suggest appropriate services. An emotion-based search engine can be made that ranks text data according to the emotion requested by the user. A negative-Emotion Alert system can be developed to avoid any mishap like, suicide, as it is the most common problem in mental illness. Based on the found emotion, a therapy recommendation system can be made to advise different types of therapy.

## Conclusion

In this paper, we built a new dataset for the persuading diseases of Pakistan including Dengue, Malaria, HIV/AIDS, Influenza/Flu, Hepatitis, and Cancer. We also add the Coronavirus disease class in the dataset as it is the gravitating disease over all the world these days. We labeled the disease distributed dataset into emotion classes comprising of Happy, Sad, Fear, Anger, Excited, and Bored. The sample documents are then pre-processed with standard text cleaning techniques. We train six supervised machine learning classifiers and evaluate their performance based on different features involving Count Vectorization and TF-IDF (Word, N-gram, and Char levels). We followed two approaches during the training of classifiers, *i.e.,* including and excluding the minority class. The obtained results are satisfactory in both scenarios. While including the minority class MLP and RF with accuracy scores 87% and 86%. Other models also give comparable results with 84% and 85% accuracy. In contrast to this, we get similar results by having RF again as the best performing model along with XGB with accuracy 86% and 84%. We also test the best model on unseen text data and achieve 82% accuracy rate. This shows sufficient and adequate working of the trained model on the textual dataset.

Moreover, we map the negative emotions to psychological health issues. We prove that a disease suffering person or patient with any negative emotion at last steers to worthless and hopeless feelings. The prediction of negative emotion reveals an alarming situation. If such emotions are poorly managed or not handled properly then chronic stress, hormonal imbalance, brain issues and disturbance of the immune system may occur.

## Future Directions

In this study, emotion detection from patient text data is performed. Predicting the emotion and filtering negative emotions are mapped to psychological disorganization. The proposed methodology can be implemented for the development of real-time web-based system. Further, the built dataset can be expanded in terms of either emotion classes or disease type. The developed emotion labeled dataset can be updated according to other target diseases with respect to some other location (here Pakistan’s prevailing diseases are considered). Some other machine learning (supervised or unsupervised) models can be tested. This work can be extended by using some embeddings and deep learning models.

## Supplemental Information

10.7717/peerj-cs.751/supp-1 Supplemental Information 1 EmoHD Dataset

## Additional Information and Declarations

### Data Availability

The following information was supplied regarding data availability: The code is available at GitHub: https://git.io/JL6tt
The EmoHD Dataset is available as a Supplemental File.
